# Schild Analysis of the Interaction between Parthenolide and Cocaine Suggests an Allosteric Relationship for Their Effects on Planarian Motility

**DOI:** 10.3390/biom14091168

**Published:** 2024-09-18

**Authors:** Jyothi Kakuturu, Mary O’Brien, Oné R. Pagán

**Affiliations:** 1 Department of Biology, West Chester University, West Chester, PA 19383, USA; jyothi91@hotmail.com; 2 MedStar Health, Columbia, MD 21044, USA; 3 Department of Public Health Sciences, Penn State College of Medicine, Hershey, PA 17033, USA; mobrien7898@gmail.com

**Keywords:** planaria, cocaine, parthenolide, motility, Schild analysis, allosteric, orthosteric

## Abstract

The freshwater planarian is an emerging animal model in neuroscience due to its centralized nervous system that closely parallels closely parallels the nervous system of vertebrates. Cocaine, an abused drug, is the ‘founding member’ of the local anesthetic family. Parthenolide, a sesquiterpene lactone, acts as a behavioral and physiological antagonist of cocaine in planarians and rats, respectively. Previous work from our laboratory showed that both parthenolide and cocaine reduced planarian motility and that parthenolide reversed the cocaine-induced motility decrease at concentrations where parthenolide does not affect the movement of the worms. However, the exact mechanism of the cocaine/parthenolide antagonism is unknown. Here, we report the results of a Schild analysis to explore the parthenolide/cocaine relationship in the planarian *Girardia tigrina*. The Schild slopes of a family of concentration–response curves of parthenolide ± a single concentration of cocaine and vice versa were −0.55 and −0.36, respectively. These slopes were not statistically different from each other. Interestingly, the slope corresponding to the parthenolide ± cocaine (but not the cocaine ± parthenolide) data set was statistically different from −1. Our data suggest an allosteric relationship between cocaine and parthenolide for their effect on planarian motility. To the best of our knowledge, this is the first study about the mechanism of action of the antagonism between cocaine and parthenolide. Further studies are needed to determine the specific nature of the parthenolide/cocaine target(s) in this organism.

## 1. Introduction

Freshwater planarians have a distinguished history in regenerative and developmental biology research. The systematic study of these organisms in this context has been ongoing for more than two centuries and is still a source of profound insights in these areas, particularly upon the availability of molecular biology, genomics, and bioinformatics tools and techniques [1,2,3,4,5,6,7]. More recently, planarians have emerged as a powerful model to study regenerative processes at the molecular level, specifically in nervous tissue (reviewed in [8,9,10,11,12]). Planarians are also a promising model system in neuroscience and pharmacology for three main reasons: (1) the presence of vertebrate-like neurotransmitter systems in these organisms, (2) the behavioral responses that planarians exhibit upon exposure to exogenous substances are closely reminiscent of the effects of drugs in vertebrates, and (3) the similarities of the planarian nervous system to the nervous system of vertebrates in terms of cellular biology and morphology [13,14,15,16,17]. An especially active area of pharmacological research using planarians is the in vivo study of the effect of abused drugs, including cocaine, amphetamines, ethanol, and nicotine, among others, to explore their effects in terms of acute exposure (toxicity) or chronic administration, meaning sub-lethal concentrations that may lead to phenomena like desensitization, tolerance, and withdrawal-like behaviors [18,19,20,21,22,23,24,25,26,27]. Based on multiple lines of evidence, planarians are uniquely positioned to integrate the ostensibly separate disciplines of regeneration, neuroscience, and pharmacology [28].

**Figure 1:**
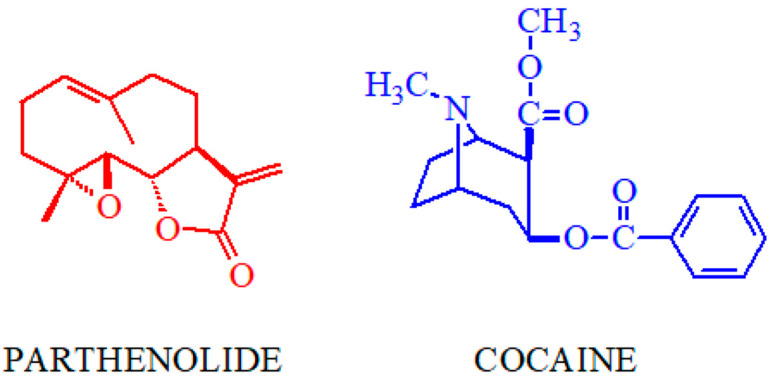
Compounds studied in this work.

Cocaine (Figure 1) is a plant-derived compound (mainly from plants of the *Erythroxylum genus* [29]) and is the parent compound of the local anesthetic superfamily, which primarily targets voltage-gated sodium channels on the surface of nerve cells [30,31]. Besides its local anesthetic properties, cocaine’s main behavioral effect in vertebrates is through its interaction with neurotransmitter transporters, particularly the dopamine transporter [32]. The naturally occurring sesquiterpene lactone parthenolide (Figure 1) is mainly isolated from the feverfew plant (*Tanacetum parthenium* [33]), a plant used in traditional medicine in various cultures. Parthenolide and other sesquiterpenoid lactones are currently in clinical trials for their antitumoral and anti-inflammatory properties, among other interesting biochemical effects. However, parthenolide and related compounds also induce contact dermatitis and genotoxic effects [34,35,36,37].

The logical thread that led our research group to the discovery and study of the interaction of cocaine and the sesquiterpene lactones of the parthenolide family and the initial use of planarians for these purposes is reviewed in detail elsewhere [4,38]. Our research established that parthenolide is a behavioral antagonist of cocaine in the planarians system, capable of counteracting the effects of acute cocaine exposure, which mimic toxicity effects [39], as well as preventing the induction of withdrawal-like behaviors by cocaine, which mimic chronic administration [40]. We also determined the minimal structural determinants of the parthenolide-like molecules capable of antagonizing the motility decrease induced by cocaine [41]. Additionally, we established that parthenolide is a specific cocaine antagonist for another acute administration effect, the induction of seizure-like movements through experiments in which parthenolide prevented the expression of seizure-like behavior by cocaine but did not prevent the expression of similar behavior induced by nicotine and amphetamines, among other drugs [42]. Furthermore, a collaboration with the group of Dr. Carlos Jiménez-Rivera (University of Puerto Rico Medical Science Campus, San Juan, PR, USA) resulted in the finding that parthenolide antagonized the inhibitory effect of cocaine on neuronal firing rate in the ventral tegmental area of rats [43], opening the door to the potential of parthenolide as a cocaine antagonist in vertebrates validating the potential usefulness and relevance of the planarian model for vertebrate studies.

Although the molecular targets of cocaine in mammals are well understood, the identity of the biochemical target(s) of parthenolide in vertebrates is less clear, and even less is known about the mechanism of action, molecular nature, and interaction of the cocaine and parthenolide target(s) in planarians and rats. In the present work, we explore the relationship between parthenolide and cocaine in terms of their effects on planarian motility using a Schild analysis to obtain information about the mechanism of this interaction.

## 2. Materials and Methods

Brown planarians (*Girardia tigrina*) were purchased from Ward’s (Rochester, NY). Parthenolide and cocaine were purchased from Tocris (Minneapolis, MN) and Sigma-Aldrich (St. Louis, MO, USA), respectively. General laboratory materials, chemicals, and supplies were purchased from Fisher Scientific (Suwanee, GA, USA). All graphs and statistical analyses were carried out with the Prism software package, version 10.3.1 (GraphPad Inc., San Diego, CA, USA). Upon arrival at our laboratory, the flatworms were transferred to artificial pond water (APW: 6 mM NaCl, 0.1 mM NaHCO_3_, 0.6 mM CaCl_2_) and allowed to acclimate to laboratory conditions for at least 24 h before using them for experiments. The worms were used within three weeks of arrival. The APW was changed at least once every day, except during weekends, and immediately before any experiments. All experiments were performed at room temperature in APW containing 0.1% dimethylsulfoxide (DMSO) as a solubility-enhancing agent for parthenolide; this concentration of DMSO does not display any evident behavioral or toxic effects in planarians [44]. The solubility of parthenolide in aqueous solvent is a pervasive problem; we used DMSO to minimize this factor in all our parthenolide-related work [39,40,41,42,43,44].

**Figure 2:**
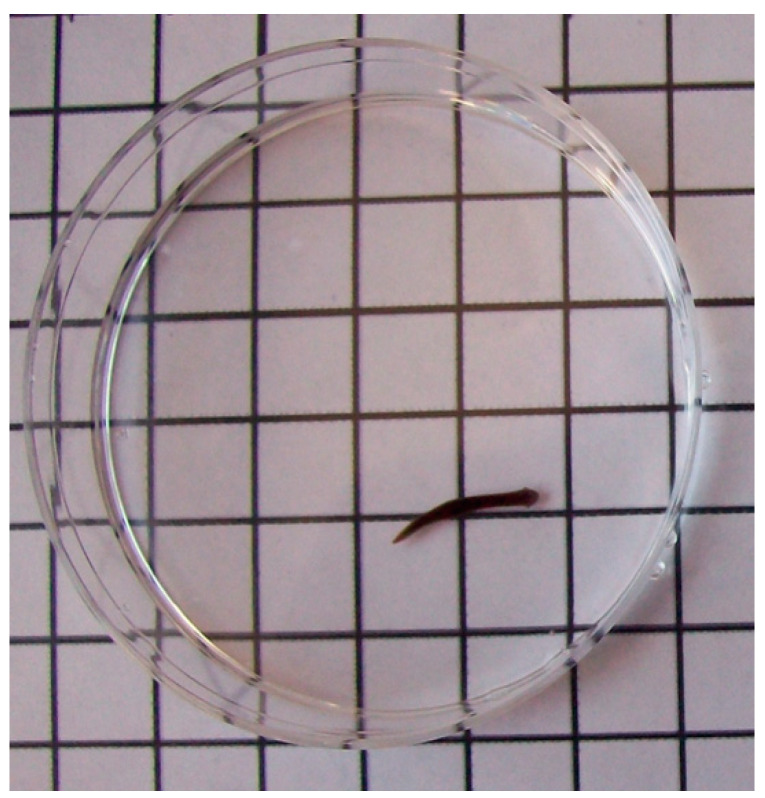
Representative experimental setup showing a planarian in a 6 cm plastic dish on a 1 cm^2^ gridline (see text). Picture taken by O.R. Pagán.

We adapted a published protocol to measure planarian motility [45], as modified in [44,46]. Briefly, using a small paintbrush or plastic pipette, a planarian (1.0–1.5 cm long) was placed in a transparent, APW-rinsed, 6 cm polystyrene Petri dish. The dish was placed over a 1 cm^2^ grid (Figure 2), and 5 mL of APW/0.1% DMSO (control) or 5 mL of the experimental solutions in 0.1% DMSO was added to the dish. The motility of the worm was measured by counting the squares crossed or recrossed per minute for 5 min, which is the exposure time of the pharmacological agents to the worms. Each worm was used only once.

The data were plotted as cumulative crosses vs. time and fit using simple linear regression to obtain its slope. In experiments where the worms were exposed to various concentrations of the experimental compounds, the slopes obtained from the linear regression were normalized to control slopes and graphed as the fraction of control vs. the experimental compound concentration [44,45,46]. To analyze these concentration–response curves, we fit the data to an empirical Hill equation (Equation (1)).

F = IC_50_^n^/(IC_50_^n^ + [Compound]^n^) (1)

F is the fraction of control, [Compound] is the experimental compound concentration in μM, the IC_50_ is the compound concentration that decreased planarian motility by 50%, and n is the Hill coefficient. The Hill coefficient is a parameter that can be used to estimate the kind and the degree of cooperativity in multimeric proteins. Although a useful parameter for the initial exploration of cooperativity, its direct link to the actual physical reality of a system is unclear, especially in the context of behavioral studies [47,48], and, therefore, the Hill coefficient is not further discussed in the present work. In general, the usefulness of empirical models lies in the fact that they may provide helpful mechanistic insights and information about the pharmacology of a compound when little or no information exists about either the ligand or its putative target [49,50].

We performed two sets of experiments to observe the effect of parthenolide or cocaine on the IC_50_ of the other to decrease planarian motility: a series of concentration–response curves of cocaine in the absence or the presence of single concentrations of parthenolide (Δ cocaine ± parthenolide), and a series of concentration–response curves of parthenolide in the absence or the presence of single concentrations of cocaine (Δ parthenolide ± cocaine). In these experiments, the compound used in the concentration–response curve (the Δ compound) was defined as ‘Compound A’, and the compound added at a single concentration was defined as ‘Compound B’. We used concentrations of Compound B that did not slow down the worms [39]. The shift in the IC_50_ for Compound A induced by the single concentration of Compound B was subjected to a Schild analysis by fitting the data to a linear equation (Equation (2)).

log (DR−1) = S (−log [B]) + b(2)

The DR is the dose ratio (the IC_50_ value obtained from a concentration–response curve of Compound A in the presence of a single concentration of Compound B over the IC_50_ value of Compound A in the absence of Compound B). S is the Schild slope, and b is the y-intercept. A Schild slope that is statistically equal to unity suggests an orthosteric relationship between the tested compounds; conversely, a Schild slope that is statistically different from unity suggests an allosteric relationship between the tested compounds [51,52,53,54].

## 3. Results

**Figure 3:**
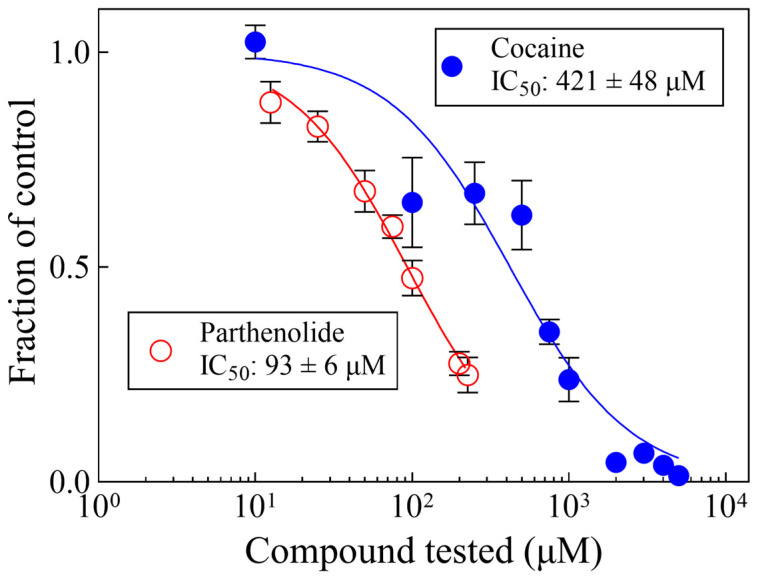
Parthenolide and cocaine decrease planarian motility in a concentration-dependent manner. Each data point represents the average of the responses of 6 worms. The lines were generated by fitting the data to Equation (1). The error bars represent the standard error of the mean. Symbols without visible error bars are bigger than the size of the SEM value.

Figure 3 shows the concentration–response curves of cocaine or parthenolide for planarian motility. The IC_50_ values (421 and 93 μM for cocaine and parthenolide, respectively) are consistent with previous reports from our laboratory [39].

**Figure 4:**
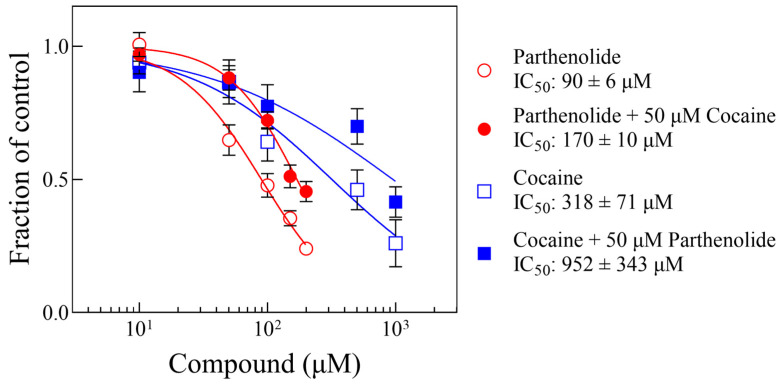
Representative concentration–response curves of motility decrease by parthenolide or cocaine in the absence and the presence of a single concentration of the other, as indicated (see text and Table 1). Each data point represents the average of the responses of 4 worms. The lines were generated by fitting que data to Equation (1). The error bars represent the standard error of the mean. The Hill and Schild parameters for all pairs are listed in Table 1. Symbols without visible error bars are bigger than the size of the SEM value. The IC_50_ values from the parthenolide and parthenolide ± cocaine are statistically different from each other (*p*-value < 0.0001), as are the IC_50_ values from the cocaine and cocaine ± parthenolide (*p*-value = 0.021).

**Figure 5:**
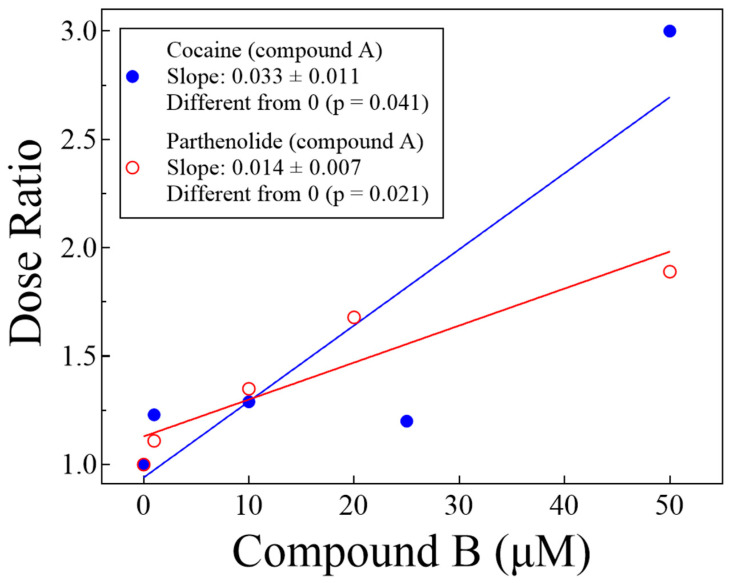
Linear regression analysis of the dose ratio (DR, from Table 1) vs. the concentration of Compound B shows that the apparent IC_50_ of Compound A (parthenolide or cocaine) increases in the presence of the other in a concentration-dependent manner (see text).

Figure 4 shows representative curve pairs of Δ parthenolide ± cocaine or Δ cocaine ± parthenolide, showing the IC_50_ shift of the experimental compounds, as indicated. Pairs of curves for each of the conditions were obtained, and their parameters from the fit to either Equation (1) or Equation (2) are shown in Table 1. Figure 5 shows a plot of the dose ratio (DR) vs. the concentration of Compound B (from Table 1), indicating that the apparent IC_50_ of Compound A (parthenolide or cocaine) increases in the presence of the other in a concentration-dependent manner.

**Figure 6:**
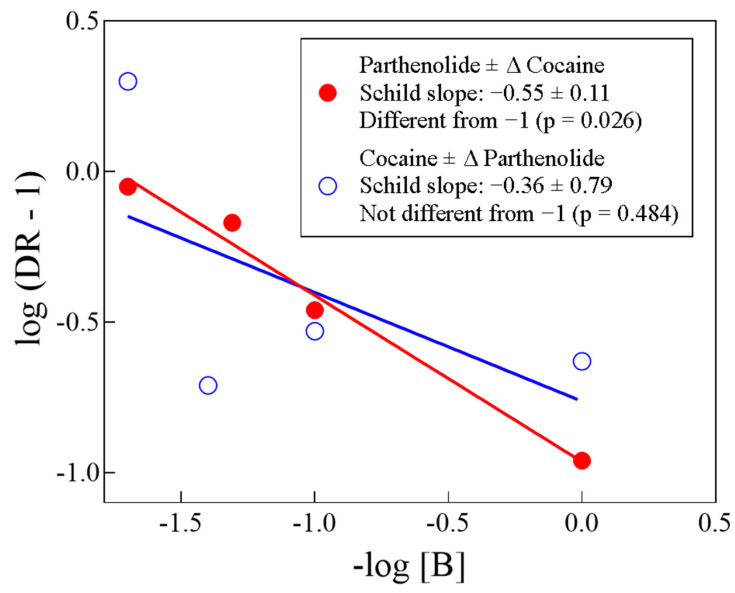
Schild plots (abscissa and ordinate values from Table 1; see text). Although the Schild slope of parthenolide ± cocaine is statistically different from −1 and the Schild slope of cocaine ± parthenolide is not statistically different from −1, the two slopes are not statistically different from each other (*p* = 0.633, unpaired t-test; see text).

Figure 6 shows Schild plots of the two tests we performed. For the Δ parthenolide ± cocaine or of Δ cocaine ± parthenolide, the Schild slopes were −0.55 ± 0.11 and −0.36 ± 0.79, respectively. Both slopes were statistically identical to each other (*p* = 0.633, unpaired t-test). However, only the Δ parthenolide ± cocaine slope (−0.55) was statistically different from −1 (*p* = 0.026), while the Δ cocaine ± parthenolide slope (−0.36) was not (*p* = 0.484), likely due to data dispersion, although a quite interesting topic for further explorations of this biochemical problem.

## 4. Discussion

In this work, we present an analysis of the parthenolide and cocaine antagonism in the planarian model. The molecular targets of cocaine in vertebrates are well known (voltage-gated sodium channels and monoamine transporters), and its interaction with these targets accounts for virtually every observed effect of cocaine in biological systems. Planarians possess genes analogous to these proteins according to genomic data available in various databases [6,7,55,56]. All available evidence suggests that these proteins function in planaria as in vertebrates. In contrast, the nature of the molecular target(s) of parthenolide and related sesquiterpene lactones is less clear; in fact, parthenolide has been described as having a “*promiscuous bioactivity profile*” [35]. Most studies on the molecular effects of parthenolide and related compounds focus on their impact on IkB kinases, the nuclear factor kappa-B (NF-κB), and several classes of cytokines downstream, which may account for parthenolide’s antitumoral and anti-inflammatory properties; parthenolide, in particular, seems to decrease NF-κB activity at the transcriptional level and by direct inhibition of IKK-B kinases [57,58].

NF-κB proteins seem to be a common target for sesquiterpene lactones and cocaine. Cocaine increases NF-κB activity in H9C2 cells (striated cardiomyocytes from rats, [59]), and mice chronically exposed to cocaine display enhanced activity of NF-κB pathways [60]. Additionally, sodium diethyldithiocarbamate trihydrate (DTT), a NF-κB inhibitor, reverses the cocaine-induced expression genes related to axon guidance pathways in mice [61]. Paradoxically, long-term cocaine exposure induces NF-κB activity in mice hippocampus [62]. Interestingly, the viral knockdown of NF-κB within the rat nucleus accumbens induces a reduction in cue-induced cocaine-seeking in males but not female rats [63], and chronic cocaine administration induces NF-κB-dependent transcription in mice [64]. There are analogs of NF-κB proteins in various planarian species [6,7,55,56]. If NF-κB is the common target of parthenolide and cocaine in the planarian model, the most likely nature of this relationship would be a common pathway rather than direct binding sites for both experimental compounds on the protein.

The fact that cocaine and parthenolide affect the effect of the other in planarian motility indicates a mutually exclusive binding relationship between these compounds for their motility decrease properties in planarians. This interpretation is consistent with previous studies from our laboratory [39] (Figure 3 and Figure 4). However, the exact nature of this relationship is unknown. The most straightforward interpretation of our data would be that parthenolide and cocaine display an orthosteric interaction (a single type of binding site shared by both compounds and, alternatively, separate yet overlapping binding sites). 

On the other hand, the parthenolide/cocaine interaction can be allosteric, where each compound would have its individual, non-overlapping (yet related to the other’s) binding site. To distinguish between these two possibilities, we performed a Schild analysis of the IC_50_ shift data from Table 1. Briefly, if the value of the Schild slope is statistically equal to unity, the data would suggest an orthosteric relationship between parthenolide and cocaine. On the other hand, if the slope is significantly different from unity, the data would suggest an allosteric relationship between the two compounds [51,52,53,54]. The results of our analysis suggest that the parthenolide/cocaine relationship for their effect on motility in the planarian model is allosteric in nature (see Section 3 “Results”).

It is important to note that in planarians, cocaine, but not parthenolide, induces other types of behaviors in addition to motility decrease. Some of these behaviors are expressed under acute exposure conditions, such as seizure-like hyperkinesia [21,25,26,39,42], dark/light environmental preference (which is oftentimes interpreted as an anxiety-like response [64,65]), and behavioral and cross-sensitization [66]. Also, chronic exposure to cocaine induces conditioned place preference behaviors [67] and even withdrawal-like behaviors [27,40]. Of these cocaine-induced behaviors, we demonstrated that parthenolide and related analogs antagonize cocaine-induced motility decrease and seizure-like hyperkinesia, as well as withdrawal-like behaviors [39,42]. It is unlikely that all these behaviors are influenced by the same proteins; further studies are needed to elucidate these interesting effects.

Regarding the chemically induced decrease in motility behavior in planarians, microtubules are another possible protein candidate that may be a common target of parthenolide and cocaine. Parthenolide and costunolide interfere with microtubule physiology [68,69,70,71], and microtubules and microtubule-associated proteins are “non-canonical” targets of cocaine [72,73,74,75]. However, planarian motility depends on properly functioning cilia, which depends on normal microtubule physiology [76,77]. Thus, the effect of parthenolide and cocaine on microtubules, in general, and cilia, in particular, is a potential area of research.

Interestingly, parthenolide seems to promote functional nervous tissue regeneration in mice [75]; this contrasts with results obtained from our laboratory that suggest that parthenolide slows down the rate of planarian brain regeneration (manuscript in preparation) using a behavioral method developed in our laboratory [78]. Additional studies that might contribute to the understanding of the parthenolide/cocaine relationship in planarians may include detailed mechanistic analyses of cocaine-induced seizure-like hyperkinesia, dark/light environmental preference, withdrawal-like behaviors, and habituation and desensitization. As discussed previously, cocaine induces such effects in planarians. These studies will help paint a clearer picture of the relationship between our experimental compounds, especially since it is likely that cocaine induces such effects by affecting different molecular targets. The study of these other cocaine-induced behaviors in planarians and their alleviation by sesquiterpene lactones of the parthenolide class will likely shed light on the mechanism of action of cocaine and perhaps other related abused drugs.

**Figure 7:**
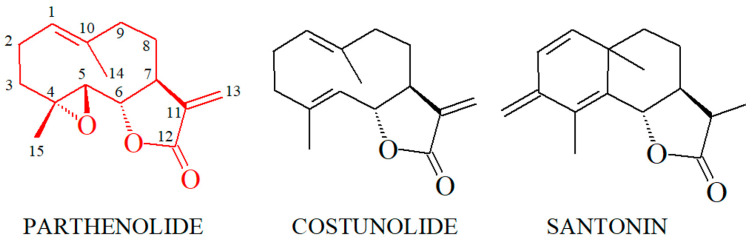
Structural comparison of parthenolide, costunolide, and santonin (see text).

Furthermore, an exciting possibility would be to conduct detailed structure–activity studies of cocaine alleviation by different sesquiterpene lactones of the parthenolide class. These studies may prove valuable for understanding this pharmacological problem. We have preliminary information in this respect. We established that a lactone moiety is necessary for an anti-cocaine effect [39,41] and that costunolide, a close structural analog of parthenolide, is about 1.6 times more potent for the alleviation of cocaine-induced motility decrease in planarians [39]. In contrast, santonin, another sesquiterpene lactone (Figure 6), does not alleviate the cocaine-induced motility decrease in planarians [39]. This information showcases the potential of structure–activity studies in the present context, as it is evident that relatively small structural differences significantly affect the potency of sesquiterpene lactones in this context, especially considering the close structural similarity between parthenolide and costunolide. The only difference between these two compounds is that a double bond substitutes the epoxy group in carbons 4 and 5 of parthenolide, while santonin displays additional structural differences compared with parthenolide and costunolide (Figure 7). Despite these structural differences in these sesquiterpene lactones, parthenolide, costunolide, and santonin still interact with their main target, the NF-κB proteins [57,58,79,80].

## 5. Conclusions

Our work confirms that parthenolide and cocaine are inhibitors of each other in the planarian system, consistent with previous reports from our laboratory [39]. Furthermore, our results suggest an allosteric relationship between these compounds based on the Schild analysis discussed earlier. Further experiments may include additional concentration–response curves of both compounds in the absence and the presence of the other, which should fine-tune our results and provide more statistical power to our experiments. We hypothesize that the most likely common target for the decrease in planarian motility by cocaine or parthenolide are microtubules, since they would be affected more directly (hence acting faster) than any possible effects in biochemical pathways such as the ones involving NF-κB proteins. Although an intriguing possibility, the apparent roles of NF-κB or microtubule proteins as links between parthenolide and cocaine are speculative at the moment. More studies are needed to explore this possibility. Any additional information that we obtain from this approach, as well as studies on other cocaine-induced behaviors in planarians and their alleviation by sesquiterpene lactones, has the potential to contribute to a better understanding of how cocaine and related compounds cause their harmful effects in vertebrates and will likely point to potential strategies to prevent such undesirable effects in vivo.

## Figures and Tables

**Table 1:** Hill and Schild parameters for the data fit to Equations (1) and (2) (see text).

**Treatment**	**IC_50_ (μM ± SEM)**	**F-Test *p*-Value**	**DR**	**log (DR-1)**	**B (Cocaine, μM)**	**−log B**
Parthenolide (no Cocaine)	127 ± 13	-	-	-	-	
Parthenolide +1 μM cocaine	141 ± 11	0.543	1.11	−0.96	1	0
Parthenolide (no Cocaine)	118 ± 13	-	-	-	-	
Parthenolide +10 μM cocaine	159 ± 24	0.236	1.35	−0.46	10	1
Parthenolide (no Cocaine)	115 ± 7	-	-	-	-	
Parthenolide +20 μM cocaine	193 ± 25	0.0001	1.68	−0.17	20	1.31
Parthenolide (no Cocaine)	90 ± 6		-	-	-	
Parthenolide +50 μM cocaine	170 ± 10	<0.0001	1.89	−0.05	50	1.70
**Treatment**	**IC_50_ (μM ± SEM)**	**F-Test *p*-Value**	**DR**	**log (DR-1)**	**B (Parthenolide, μM)**	**−log B**
Cocaine (no Parthenolide)	158 ± 25	-	-	-	-	
Cocaine +1 μM Parthenolide	195 ± 23	0.526	1.23	−0.63	1	0
Cocaine (no Parthenolide)	293 ± 37	-	-	-	-	
Cocaine +10 μM Parthenolide	379 ± 59	0.443	1.29	−0.53	10	1
Cocaine (no Parthenolide)	224 ± 33	-		-	-	
Parthenolide +25 μM cocaine	268 ± 70	0.247	1.20	−0.71	25	1.40
Cocaine (no Parthenolide)	318 ± 71	-		-	-	
Cocaine +50 μM Parthenolide	952 ± 343	0.021	3.0	0.30	50	1.70

## Data Availability

Available upon request.
